# Simultaneous Detection of Nine Key Bacterial Respiratory Pathogens Using Luminex xTAG^®^ Technology

**DOI:** 10.3390/ijerph14030223

**Published:** 2017-02-24

**Authors:** Luxi Jiang, Hongyu Ren, Haijian Zhou, Tian Qin, Yu Chen

**Affiliations:** 1 State Key Laboratory for Infectious Disease Prevention and Control, National Institute for Communicable Disease Control and Prevention, Chinese Center for Disease Control and Prevention, Beijing 102206, China; jlx67@126.com (L.J.); renhongyu@icdc.cn (H.R.); zhj_0901@163.com (H.Z.); 2 Department of Respiratory Medicine, Shengjing Hospital of China Medical University, Shenyang 110004, China; 3 Department of Respiratory Medicine, Zhejiang Provincial People’s Hospital, Hangzhou 310014, China

**Keywords:** respiratory pathogens, lower respiratory tract infections, multiplex PCR, Luminex, detection method

## Abstract

Early diagnosis and treatment are crucial to the outcome of lower respiratory tract infections (LRTIs). In this study, we developed an assay combining multiplex PCR and Luminex technology (MPLT) for the detection of nine important respiratory bacterial pathogens, which frequently cause LRTIs. These were *Streptococcus pneumoniae*, *Moraxella catarrhalis*, *Staphylococcus aureus*, *Streptococcus pyogenes*, *Haemophilus influenzae*, *Mycoplasma pneumoniae*, *Legionella* spp., *Pseudomonas aeruginosa*, and *Klebsiella pneumoniae*. Through the hybridization reaction between two new synthesized multiplex PCR products and MagPlex-TAG Microspheres, we demonstrate that the detection limits for these nine pathogens were as low as 10^2^–10^3^ CFU/mL. Furthermore, 86 clinical bronchoalveolar lavage fluid specimens were used to evaluate this method. Compared with the results of nine simplex real-time PCR reactions targeting these nine pathogens, this MPLT assay demonstrated a high diagnostic accuracy for *Streptococcus pneumoniae* (sensitivity, 87.5% and specificity, 100%). Furthermore, sensitivity and specificity for the other eight pathogens all attained 100% diagnostic accuracy. In addition, the consistency between MPLT and the nine real-time PCR reactions exceeded 98.8%. In conclusion, MPLT is a high-throughput, labor-saving and reliable method with high sensitivity and specificity for identifying nine respiratory pathogens responsible for LRTIs. Indeed, this assay may be a promising supplement to conventional methods used to diagnose LRTIs.

## 1. Introduction

Lower respiratory tract infections (LRTIs) are caused by numerous pathogens and, if untreated, these can progress to severe infections and even death. As reported by The Global Burden of Disease Study, LRTIs were the second-leading cause of deaths in 2013 [1]. The age-standardized mortality rate of LRTIs has been reported to be 41.7 per 100,000 (95% CI 37.1–44.1 per 100,000) [2]. The elderly and people with weakened immune systems are particularly susceptible to these types of infections [3].

A large and increasing number of pathogens, including bacteria, atypical agents and viruses, are responsible for LRTIs [4,5,6]. The clinical presentations and radiology of LRTIs caused by different pathogens are very similar, and this makes it difficult to differentiate these pathogens using only clinical symptoms and lung imaging. The identification of pathogens in severe LRTIs would be of great value in facilitating prompt and successful medical treatment. Therefore, a powerful assay that can simultaneously detect multiple pathogens both quickly and accurately is urgently needed.

Traditional methods used to identify respiratory pathogens include microbial culture, staining methods, urinary antigen tests, and serological tests [7]. These traditional methods can help identify respiratory pathogens to some extent. However, they usually have particular disadvantages: staining methods have poor sensitivity, culture of pathogens is time-consuming, and the specialized culture media for atypical agents and viruses are not accessible in most hospitals and clinical institutions. Serological responses may occur late, thus requiring paired sera at least a week apart, and such serological assays are commonly used in retrospective diagnosis and systematic surveys [8,9]. 

Molecular methods such as polymerase chain reaction (PCR) and real-time PCR offer high sensitivity and specificity, and have been generated for the rapid identification of respiratory pathogens [10,11,12]. These methods can be used to analyze clinical samples, including sputum, bronchoalveolar lavage fluid (BALF) and blood. However, the throughput of most molecular methods is relatively limited, and most molecular methods cannot detect more than five pathogens in one reaction [10,12,13,14]. Therefore, designing a high-throughput, labor-saving, and rapid method to identify and differentiate respiratory pathogens would be important in enhancing rapid response for the prompt treatment of LRTIs.

Compared with other methods, the Luminex xTAG^®^ technology is a high-throughput, rapid, labor-saving multiplex assay offering both high sensitivity and specificity. This technology is reported to detect up to 100 different targets in a single reaction vessel simultaneously [15,16,17]. This technology is also very useful because of its flexibility in detecting a broad range of pathogens, which means it can be organized into different multiplex assays for different diagnostic targets, as required.

In this study, we aimed to develop a high-throughput and useful multiplex molecular method for the rapid detection of respiratory bacteria of public health importance. We developed an assay combining multiplex PCR and Luminex xTAG^®^ technology (MPLT) for detection of nine key respiratory bacteria, including *Streptococcus pneumoniae*, *Moraxella catarrhalis*, *Staphylococcus aureus*, *Streptococcus pyogenes*, *Haemophilus influenzae*, *Mycoplasma pneumoniae*, *Legionella* spp., *Pseudomonas aeruginosa*, and *Klebsiella pneumoniae*. These pathogens account for most of the respiratory bacteria that precipitate LRTIs [3]. The sensitivity and specificity of the MPLT assay was evaluated, and 86 clinical BALF specimens from LRTI patients were assayed for simultaneous bacteriological detection using the MPLT assay. Using this new method, we compared the results with the data from the nine individual real-time PCRs.

## 2. Materials and Methods

### 2.1. Ethics

Ethical approval for this study was obtained from the Institutional Review Boards of the Shengjing Hospital of China Medical University, China (2016PS238K).

### 2.2. Bacterial Strains

The nine target respiratory bacterial pathogens used in this study are listed in Table 1 (numbered 1 to 9). The other four respiratory bacteria listed in Table 1 (numbered 10 to 13) were used as negative control organisms for the evaluation of specificity. All bacterial organisms and DNA extracts were stored at −80 °C until they were tested.

### 2.3. Clinical Specimens

A total of 86 clinical BALF specimens were collected from 2014 to 2016 at two hospitals in China. All specimens were obtained from patients with LRTI, including those with pneumonia, acute bronchitis, capillary bronchitis, or other respiratory tract infections. A total of 86 clinical BALF specimens were gathered from 86 individual patients. The following criteria were used to diagnose LRTI: presence of fever and/or an increased leukocyte count (≥11 × 10^9^ /L), increased focal symptoms from the lower airways, with at least one of the following three incident symptoms: increase in dyspnea, coughing, and/or purulent sputum [18]. All clinical BALF specimens were stored at −80 °C until they were tested.

### 2.4. DNA Extraction

The DNA of 11 bacteria (13 bacteria in Table 1 except for *M. pneumoniae and M. tuberculosis complex*) was extracted using a QIAamp DNA Mini Kit (Qiagen, Hilden, Germany), according to the manufacturer’s instructions. The 8 bacterial specimens (9 target respiratory pathogens except *M. pneumoniae*) were serially diluted ten-fold from 10^8^ to 10^0^ CFU/mL, and DNA concentrations were measured by NanoDrop 2000 (Thermo Fisher Scientific, Shanghai, China). The DNA extracts of *M. pneumoniae* were also diluted ten-fold from 10^1^ ng to 10^−6^ ng.

The clinical BALF specimens comprised a volume of 1–2 mL, and were centrifuged at 8000 rpm for 10 min; one part of the sediment (200 μL) was used for DNA extraction, which was carried out using the QIAamp blood kit (Qiagen) according to the manufacturer’s instructions (Qiagen). The DNA was eluted in a final volume of 100 μL and stored at −20 °C.

### 2.5. Primers for Pathogen Amplification

Nine sets of primers were used in this assay. The primers for these nine pathogens were selected from previously published studies, and primers were selected on the basis of having similar melting temperatures so that simultaneous amplification using the same conditions could occur for all multiplex reactions. The expected amplicon sizes were between 94 and 650 bp. It is commonly known that small amplicons provide improved signal intensity and hybridization efficiency in the MPLT system [16,17].

Each forward primer targeting the nine pathogens was modified by a unique 24-base oligonucleotide “TAG” sequence at the 5’ terminus and was used to connect with the MagPlex-xTAG Microsphere (Luminex Corporation, Toronto, ON, Canada). The MagPlex-xTAG Microspheres are MagPlex beads (6.5-micron superparamagnetic beads) where each bead region is covalently pre-coupled with the 24-base oligonucleotide “anti-TAG” sequence (complementary to the “TAG” sequence). A 12-carbon amine was incorporated between the “TAG” sequence and primer. All reverse primers were biotinylated at the 5′ terminus. The primer sequences, their target genes, and the size of the resulting amplicons are shown in Table 2. All primers were synthesized by Tsingke (Beijing, China).

### 2.6. Multiplex PCR (mPCR) Amplification

First, nine sets of primers targeting the nine target bacterial organisms were confirmed by the simplex PCR reactions. The mPCR was affected by many factors such as annealing time, annealing temperature, and primer concentrations. These conditions were optimized for the mPCR to ensure specific amplification of the selected targets. Finally, two mPCR reactions were generated. 

The first multiplex PCR amplification was performed in a total volume of 30 µL containing the following: 15 μL of Premix *Taq*^TM^ (Takara Bio Inc., Shiga, Japan); 2 µL genomic DNA template; 1 μL of each primer (Table 2) for *S. pneumoniae*, *M. catarrhalis*, *S. aureus*, and *S. pyogenes*; and 5 μL of distilled water. The mPCR amplification conditions were as follows: denaturation at 95 °C for 10 min; followed by 35 cycles of 95 °C for 30 s, 54 °C for 45 s, and 72 °C for 1 min; a final extension at 72 °C for 10 min was performed. PCR reactions were performed in a ThermoCycler. The reaction products were stored at 4 °C.

The reaction volume of the second mPCR was also 30 µL and it contained 2 µL of genomic DNA template; 15 µL of Premix *Taq*^TM^ (TaKaRa Taq^TM^ Version 2.0); 1 µL of each primer for *H. influenzae*, *M. pneumoniae*, *Legionella* spp., *P. aeruginosa and K. pneumoniae*; and 3 µL of distilled water. The mPCR cycling parameters were as follows: denaturation at 95 °C for 10 min; followed by 35 cycles of 95 °C for 30 s, 60 °C for 45 s, and 72 °C for 1 min; a final extension at 72 °C for 10 min was performed. PCR reactions were performed in a ThermoCycler. The reaction products were stored at 4 °C.

### 2.7. PCR Product Identification

To examine the amplified DNA, the reaction products were resolved on a 2.0% agarose gel stained with GoldView, visualized under UV light, and analyzed using a Gel Doc system (BioRad, Hercules, CA, USA). The target gene fragments for *S. pneumoniae*, *M. catarrhalis*, *S. aureus*, *S. pyogenes*, *H. influenzae*, *M. pneumoniae*, *Legionella* spp., *P. aeruginosa*, and *K. pneumoniae* were 350 bp, 140 bp, 270 bp, 93 bp, 296 bp, 209 bp, 650 bp, 504 bp, and 364 bp, respectively.

### 2.8. Luminex Assay

The PCR-Luminex assay procedure can be read on the Luminex Corporation website (http://info.luminexcorp.com/download-the-xmap-cookbook). The assay was conducted according to these instructions.

The amplified sequences were labeled with the fluorescent reporter and hybridization to MagPlex-TAG Microspheres, which were pre-coupled with “anti-TAG” sequences. A bead mixture consisted of 2500 Microspheres of each set, per reaction. The working MagPlex-xTAG Microsphere mixture was prepared by diluting microspheres to 125 of each microsphere per set per μL in 1× Tm Hybridization Buffer (0.2 M NaCl, 0.1 M Tris, 0.08% Triton X-100, pH 8.0, filter sterilized) and was then vortexed for 20 s. The pink indicator was composed of diluting Streptavidin, R-Phycoerythrin Conjugate (SAPE, Invitrogen Corporation, Carlsbad, CA, USA) to 10 μg/mL in 1× Tm Hybridization Buffer. For each reaction, 5 μL of each amplified PCR product or distilled water, 75 μL of pink SAPE solution, and 20 μL of the MagPlex-TAG Microsphere mixture were added together before mixing in a 100 μL reaction tube (Applied Biosystem Corporation, Foster City, CA, USA). Finally, the MagPlex-TAG Microsphere-PCR product-SAPE mixtures were incubated and hybridized in a thermocycler for 30 min at 40 °C. After the hybridization reaction, the products were analyzed on the Luminex 200 analyzer (Luminex Corporation, Toronto, ON, Canada) according to the system manual. The sample size was set to 100 µL with a minimum of 100 beads per target to be analyzed. 

### 2.9. MPLT Data Analysis

The data analyzed by the Luminex xPONENT software, version 3.1 (Luminex Corporation) were reported as median fluorescence intensity (MFI). The cut-off values for nine target pathogens were obtained from the mean MFIs of 46 no-template controls run during the study period, and the determination of cut-off values were also referred to from previous studies [30,31,32]. A corrected MFI (cMFI) which was normalized to background fluorescence were defined as follows: cMFI = [MFI (sample) − MFI (background)]/MFI (background). Additionally, the cMFI values of samples were shown as the mean ± standard error of the mean (SEM). 

### 2.10. The Evaluation of Sensitivity

To determine the sensitivities of the MPLT assay, serial ten-fold dilutions (10^8^–10^0^ CFU/mL) of the 8 target bacterial DNA samples (9 target bacteria except for *M. pneumoniae*) with known starting concentrations were extracted. The *M. pneumoniae* DNA was diluted to different concentrations from 10^2^ ng/μL to 10^−6^ ng/μL. For all DNA samples in this study, they were used as the templates. The sensitivity results were confirmed in triplicate.

### 2.11. The Evaluation of Specificity

The bacterial species used to assess the specificity of the assay are listed in Table 1. For each pathogen, monoplex PCR assays were performed with its own target DNA, and negative controls (other DNA excepting the target template) were simultaneously tested for each organism. To determine the specificity of the MPLT assay, DNA from the 9 bacteria were analyzed in the two mPCR reactions and after amplification, the 9 mPCR products (5 μL each) were mixed with the MagPlex-TAG Microsphere and SAPE mixtures, before further analysis on the Luminex 200 analyzer. 

### 2.12. Detection of DNA from Clinical BALF Samples

A total of 86 BALF samples were collected from two hospitals in China. DNA was extracted from the samples and tested by both the MPLT assay and the single real-time PCR assays.

### 2.13. Real-Time PCR for Bacterial Detection

The 9 pairs of specific primers and probes used in 9 single real-time PCR reactions are shown in Table 3. We used a Stratagene Mx3000P PCR system (Stratagene, La Jolla, CA, USA) for the real-time PCR assay. The reaction system comprised 12.5 μL of Premix Ex *Taq* (Takara) and 1.25 μL of each primer and fluorescent probe. The final volume of the system was adjusted to 25 μL with 0.5 μL of ROX Reference Dye II (Takara) and distilled water. The specificities of the 9 real-time PCR reactions have been confirmed in previous studies [33,34,35,36,37,38], and the relevant references are listed in Table 3. The analytical sensitivities for these 9 pathogens were measured by ten-fold serial dilutions (10^8^–10^0^ CFU/mL) of the 8 target bacterial DNA samples (9 target bacteria except for *M. pneumoniae*), and the *M. pneumoniae* DNA diluted from 10^2^ ng/μL to 10^−6^ ng/μL. The threshold cycle (CT) values of all the dilutions for these 9 pathogens were confirmed in triplicate. All the reaction systems, amplification conditions and results of these nine real-time PCR assays are listed in Supplementary Materials.

### 2.14. Statistical Analyses

Consistency between the results of the MPLT assay and real-time PCR assays was verified using Cohen’s kappa test in SPSS software, version 17.0 (IBM SPSS, Armonk, NY, USA). The kappa value was graded as follows: <0, no agreement; 0–0.20, slight; 0.21–0.40, fair; 0.41–0.60, moderate; 0.61–0.80, substantial; and 0.81–1, almost perfect agreement.

## 3. Results

### 3.1. Specificity of the Two mPCR Assays and the MPLT Assay

**Figure 1:**
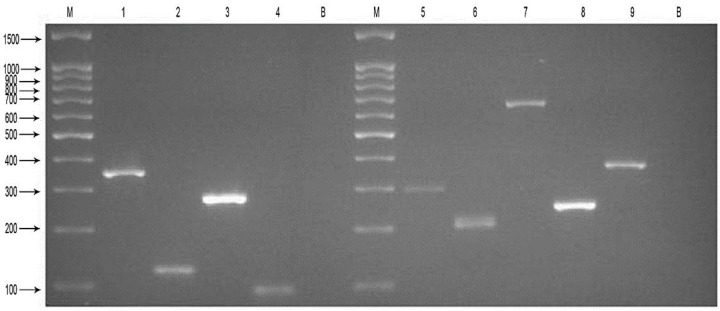
Results of the two mPCR reactions for nine target pathogens. Agarose gel electrophoresis showing the results of the four-plex PCR amplification and five-plex PCR amplification. Each DNA sample was successfully amplified and lanes are as follows: M, DNA Marker 100; 1, *S. pneumoniae* 350 bp; 2, *M. catarrhalis* 140 bp; 3, *S. aureus* 270 bp; 4, *S. pyogenes* 93 bp; B, negative control (distilled water); M, DNA Marker 100; 5, *H. influenzae* 296 bp; 6, *M. pneumoniae* 209 bp; 7, *Legionella* spp. 650 bp; 8, *P. aeruginosa* 249 bp; 9, *K. pneumoniae* 364 bp; B, negative control (distilled water).

**Figure 2:**
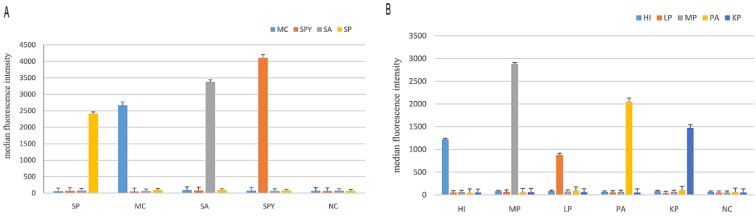
Results of the MPLT assay for nine target pathogens. (**A**) Detection of target products by the four-plex PCR-Luminex showed a high specificity. Distilled water was used as the negative control (NC). Each bar represents the average median fluorescence intensity (MFI) of triplicate samples. The error bars indicate standard deviations. SP, *S. pneumoniae*; MC, *M. catarrhalis*; SA, *S. aureus*; SPY, *S. pyogenes*; (**B**) Detection of target products by the five-plex PCR-Luminex showed a high specificity. Distilled water was used as the negative control (NC). Each bar represents the average MFI of triplicate samples. The error bars indicate standard deviations. HI, *H. influenzae*; MP, *M. pneumoniae*; LP, *Legionella* spp.; PA, *P. aeruginosa*; KP, *K. pneumonia*.

First, nine sets of specific primers and DNA templates extracted from thirteen respiratory pathogens were used and the specificity of nine primers was confirmed using nine simplex PCR reactions. Then, two mPCR reactions were generated. The resultant amplicon sizes from the two mPCR reactions for the pathogens were similar to their corresponding target sequences in Table 2. The results (Figure 1) showed that these two mPCR reactions were feasible and could achieve effective amplification for nine target respiratory pathogens with no cross-reactions being observed. The MPLT assay for each target pathogen (Figure 2A,B) showed a single and specific fluorescence signal. Cross hybridization and non-specific hybridization between sequences was not observed.

### 3.2. Sensitivity of the MPLT Assay

**Figure 3:**
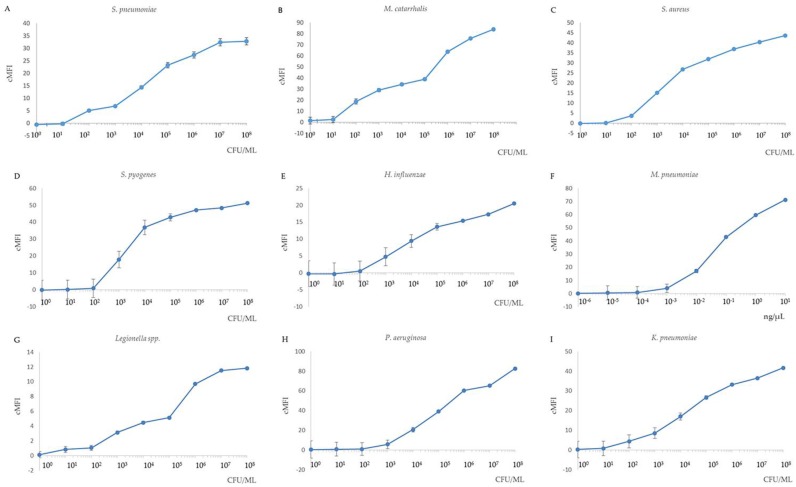
Sensitivity of the nine target bacteria in MPLT assay. Correlation of *Ct* values derived from the real-time PCR assay and different bacterial concentration. (**A**) *S. pneumoniae*; (**B**) *M. catarrhalis*; (**C**) *S. aureus*; (**D**) *S. pyogenes*; (**E**) *H. influenzae*; (**F**) *M. pneumoniae* (DNA); (**G**) *Legionella* spp.; (**H**) *P. aeruginosa*; (**I**) *K. pneumoniae*. Cut-off cMFI = 3.0. All samples were performed in triplicate. If the results from a duplicate sample analysis exceeded the cut-off cMFI, the sample was defined as MPLT-positive.

The sensitivity of the MPLT assay was examined by testing serial ten-fold dilutions of the nine target bacterial DNA samples, and distilled water was used as negative control. The detection limits in the MPLT assay are demonstrated in Figure 3A–I. The results showed that the limit of detection was 10^2^ CFU/mL for *S. pneumoniae*, *M. catarrhalis*, *S. aureus* and *K. pneumoniae* and 10^3^ CFU/mL for *S. pyogenes*, *H. influenzae*, *Legionella* spp. and *P. aeruginosa*. Finally, the detection limit for *M. pneumoniae* was 10^−3^ ng/μL.

### 3.3. Application to Clinical Samples

A total of 86 clinical BALF specimens were analyzed using the MPLT assay on the Luminex 200 analyzer. The results were confirmed by the simplex real-time PCR for the nine pathogens. The MPLT assay successfully detected and differentiated between all nine target respiratory pathogens. Compared with the results of the simplex real-time PCR, the consistency of MPLT assay exceeded 98.8% for all nine target pathogens (Table 4).

For *S. pneumoniae*, 8 of 86 clinical BALF samples were considered to be positive by MPLT assay, while 7 of 86 clinical BALF samples were detected as positive by the simplex real-time PCR assay. Furthermore, the sample which was considered to be positive by the MPLT assay but negative by the real-time PCR assay was then confirmed as a true positive by 16S rDNA sequence analysis. Coinfections were identified in 10 samples for both MPLT assay and real-time PCR assay.

The MPLT assay and the simplex real-time PCR assay both identified the same number of positive samples for *S. aureus*, *H. influenzae*, *M. pneumonia*, *P. aeruginosa* and *K. pneumoniae*.

The obtained sensitivity and specificity of this MPLT assay relative to those of real-time PCR were as follows: 87.5% and 100% for *S. pneumoniae*, and 100% and 100% for *S. aureus*, *H. influenzae*, *M. pneumonia*, *P. aeruginosa* and *K. pneumoniae*, respectively.

## 4. Discussion

*S. pneumoniae*, *M. catarrhalis*, *S. aureus*, *S. pyogenes*, *H. influenzae*, *M. pneumoniae*, *Legionella* spp., *P.* aeruginosa, and *K. pneumoniae* are the most commonly reported causative respiratory pathogens in LRTI worldwide [2,3,4,5,6]. All these bacteria have similar clinical features, and may cause multiple infections, which may increase their morbidity. Etiologic diagnosis of the causative pathogen for LRTI can pose problems, with causative organisms identified in approximately 50% of community-acquired pneumonia (CAP) cases [39,40,41]. Indeed for LRTI, the proportion of identified organisms is even lower. This is mainly due to difficulties in generating efficient associated methods that have both high sensitivity and good specificity for detection of these pathogens.

The Luminex xTAG^®^ platform is a rapid, accurate and high-throughput nucleic acid detection method which permits multiplex PCR analysis, therefore it is both attractive and reproducible. Furthermore, this method can be expanded to include more pathogens, further increasing the potential ability of the diagnostic platform. Recently, several commercial kits have been developed based on this technology. For example, the Luminex xTAG^®^ Gastrointestinal Pathogen Panel (xTAG^®^ GPP) can detect the 15 most common gastrointestinal pathogens and toxins in a single assay [42]. The Luminex xTAG^®^ Cerebrospinal Fluid Pathogens Bacterial Panel (xTAG^®^ CSF Bacteria) and the Luminex xTAG^®^ Cerebrospinal Fluid Pathogens Viral Panel (xTAG^®^ CSF Virus) can be used to detect common bacterial and viral pathogens from cerebrospinal fluid [30,31]. The Luminex xTAG^®^ Bloodstream Pathogen Panel (xTAG^®^ BPP) can be used to detect 23 pathogens commonly identified in bloodstream infections [43]. Previously, the Luminex xTAG^®^ technology has been successfully applied to simultaneously detect 18 respiratory viral pathogens (xTAG^®^ RVP FAST v2) [44]. However, there are still no commercially-available kits to detect respiratory bacterial pathogens. Recently, a Luminex assay for the detection of pathogens in acute respiratory tract infections has been built [45], however the target respiratory bacterial pathogens for this assay are limited. In addition to *S. pneumoniae*, *S. aureus*, *M. pneumoniae*, *P. aeruginosa*, and *K. pneumoniae* mentioned in this assay, we also involved *M. catarrhalis*, *S. pyogenes*, *Legionella* spp., *H. influenzae* as our target respiratory bacterial pathogens. Now, we have successfully generated this MPLT assay to investigate nine bacterial pathogens responsible for LRTI using this Luminex xTAG^®^ technology.

Other analysis platforms, such as the flow-based microarray analysis platform MCR 3 [46] and stopped polymerase chain reaction combined with chemiluminescence flow-through DNA microarray analysis platform [47], have been built. Compared with our Luminex platform, all these platfoms are characterized by low limits of detection, high reproducibility and reduced assay time [46,47]. Although multiplex real-time PCR is a direct format and is cheaper than Luminex technology, the throughput of it is low. Furthermore, due to the limitation of its fluorescence, it can detect only a limited number of bacterial pathogens in one reaction [10,12,13,14].

The results of our study showed that this MPLT assay had high specificity, sensitivity and excellent consistency compared to the real-time PCR reaction. Furthermore, we found the detection limits for these nine pathogens were as low as 10^2^–10^3^ CFU/mL (10^−3^–10^−4^ ng/μL). These results strongly suggest that the development of the MPLT assay for identification of these nine pathogens is both feasible and useful.

The sensitivity and specificity of Luminex technology is closely related to primers [48,49,50,51], and good, specific primers can avoid non-specific amplification while ensuring stability of the assay. Consequently, the primers used in our assay were carefully selected from different primers, according to the GC content of the primers and the annealing temperature. In addition, the results of the MPLT assay also showed that all primers were chosen appropriately and the nine target pathogens could be detected effectively. 

A previous study [52] showed that a ten-plex PCR-coupled liquid bead array system had lower detection limits than a single PCR. Therefore, it was important in this study to optimise the conditions of the multiplex PCR reactions. To ensure the specificity and sensitivity of this developed MPLT assay, we also optimized the hybridization reaction conditions and confirmed that 40 °C was the optimal hybridization temperature, while 30 min appeared to be the optimal hybridization time. Furthermore, proper MagPlex-TAG Microspheres were chosen for this assay, which appear to be an essential constituent of this diagnostic platform.

A total of 86 BALF samples collected over three years from patients with LRTIs were investigated by MPLT, and we found that eight samples were diagnostically positive for *S. pneumoniae*, 11 samples were positive for *S. aureus*, eight samples were positive for *H. influenzae*, one sample was positive for *M. pneumoniae*, 10 samples were positive for *P. aeruginosa* and five samples were positive for *K. pneumoniae*. Of these diagnoses, most of the results (42/43 (97.7%)) obtained from the MPLT method were consistent with those from real-time PCR assay. The only conflicting result, which was MPLT-positive but negative by the real-time PCR assay, was eventually confirmed as a true positive by 16S rDNA sequence analysis. From these data, we demonstrated that the MPLT assay had a high diagnostic accuracy for *S. pneumoniae*, *S. aureus*, *M. catarrhalis*, *S. pyogenes*, *H. influenzae*, *M. pneumoniae*, *Legionella* spp., *P. aeruginosa* and *K. pneumoniae*, respectively. The MPLT assay could simultaneously detect nine pathogens using a quick, high-throughput assay. 

The limitation of the MPLT assay is that the cost of MagPlex-TAG Microspheres and Luminex equipment used in this MPLT assay is relatively high. Although the initial outlay is expensive, this assay might be economical by ensuring prompt and effective treatment of LRTIs before they develop into serious diseases. Despite the limitation, the MPLT assay is a high-throughput method that can detect a wide range of pathogens and is easy to operate with high reliability. Based on the findings of the present study, we believe this is a promising method with potential use in the clinical setting in identifying respiratory pathogens that frequently precipitate LRTI.

## 5. Conclusions

We have established a high-throughput, labor-saving, rapid (3 h) and reliable MPLT assay with high sensitivity and specificity for the identification of nine common respiratory pathogens responsible for LRTIs. This study gives an insight into a diagnostic platform that could be a promising adjunct to the conventional and molecular methods for diagnosing LRTIs.

## Figures and Tables

**Table 1:** List of bacterial pathogens used in this study.

Number	Species	Strain ID and Description
1	*Streptococcus pneumoniae*	ATCC 49619
2	*Haemophilus influenzae*	L3 (clinical isolates from China)
3	*Moraxella catarrhalis*	ATCC 25238
4	*Pseudomonas aeruginosa*	ATCC 27853
5	*Klebsiella pneumoniae*	A915 (clinical isolates from China)
6	*Staphylococcus aureus*	ATCC 25923
7	*Legionella pneumophila*	ATCC 33152
8	*Streptococcus pyogenes*	ATCC 19615
9	*Mycoplasma pneumonia* *	ATCC 29342
10	*Escherichia coli*	ATCC 25922
11	*Bordetella pertussis*	ATCC 9797
12	*Corynebacterium diphtheriae*	CMCC 38106
13	*Mycobacterium tuberculosis complex* *	H37R

* *Mycobacterium tuberculosis complex* (20.1 ng/μL) and *M. pneumoniae* (15.5 ng/μL) were supplied as DNA extracts by the Chinese Center for Disease Control and Prevention.

**Table 2:** List of target bacteria, genes, primers, and their amplicon sizes.

Organism	Target Gene	No. of Beads Coupled with “Anti-TAG” Sequences	Sequences (5′-3′)	Working Concentration (μM)	Length (bp)	Detection Limits	Source
*S. pneumoniae*	*cpsA*	21	F-TCAAACTCTCAATTCTTACTTAAT-12C-ACGCAACTGACGAGTGTGAC R-biotin-GATCGCGACACCGAACTAAT	0.3	350	0.1 pg	[19]
*M. catarrhalis*	*16SrRNA*	8	F-AAATAACTCACTATTTCACTTACA-12C-TTGGCTTGTGCTAAAATATC R-biotin-GTCATCGCTATCATTCACCT	0.3	140	0.1 pg	[20]
*S. aureus*	*nuc*	12	F-CATAATCAATTTCAACTTTCTACT-12C-GCGATTGATGGTGATACGGTT R-biotin-AGCCAAGCCTTGACGAACTAAAGC	0.3	270	0.1 pg	[21,22]
*S. pyogenes*	*dnaseB*	9	F-CACATCTAATACTTTATACAATTC-12C-TGATTCCAAGAGCTGTCGTG R-biotin-TGGTGTAGCCATTAGCTGTGTT	0.3	93	0.1 pg	[23]
*H. influenzae*	*p6*	8	F-AAATAACTCACTATTTCACTTACA-12C-TTGGCGGWTACTCTGTTGCT R-biotin-TGCAGGTTTTTCTTCACCGT	0.3	296	0.1 pg	[24]
*M. pneumoniae*	*P1*	12	F-CATAATCAATTTCAACTTTCTACT-12C-GCCACCCTCGGGGGCAGTCAG R-biotin-GAGTCGGGATTCCCCGCGGAGG	0.3	209	1 pg	[25,26]
*Legionella* spp.	*16SrRNA*	9	F-CACATCTAATACTTTATACAATTC-12C-AAGATTAGCCTGCGTCCGAT R-biotin-GTCAACTTATCGCGTTTGCT	0.3	650	1 pg	[27]
*P. aeruginosa*	*oprI*	14	F-AATTTCTTCTCTTTCTTTCACAAT-12C-ATGAACAACGTTCTGAAATTCTCTGCT R-biotin-CTTGCGGCTGGCTTTTTCCAG	0.3	249	1 pg	[28]
*K. pneumoniae*	*Mdh*	15	F-TACTTCTTTACTACAATTTACAAC-12C-GCGTGGCGGTAGATCTAAGTCATA R-biotin-TTCAGCTCCGCCACAAAGGTA	0.3	364	0.1 pg	[12,29]

**Table 3:** Real-time PCR assays for detection of nine respiratory pathogens.

Organism	Gene Target	Oligonucleotide/Oligo Sequence	Reference
*S. pneumoniae*	*lytA*	Forward: ACGCAATCTAGCAGATGAAGCA Reverse: TCGTGCGTTTTAATTCCAGCT Probe: FAM-GCCGAAAACGCTTGATACAGGGAG-BHQ1	[33]
*M. catarrhalis*	*copB*	Forward: CGTGCGTGTTGACCGTTTTGACTTTA Reverse: CACGCTGCCAAAAATAACTGCCAAAG Probe: FAM-CAGCGGTAACCTAATCTATGCCACTC-BHQ1	[34]
*S. aureus*	*spa*	Forward: TACATGTCGTTAAACCTGGTG Reverse: TACAGTTGTACCGATGAATGG Probe: FAM-CGCGATCCAAGAACTTGTTGTTGATAAGAAGCAACCGATCGCG-BHQ1	[35,36]
*S. pyogenes*	*16SrRNA*	Forward: GAGAGACTAACGCATGTTAGTA Reverse: TAGTTACCGTCACTTGGTGG Probe: FAM-CGCGATCGCGACGATACATAGCCGACCTGGATCGCG-BHQ1	[37]
*H. influenzae*	*16SrRNA*	Forward: TTGACATCCTAAGAAGAGCTCAGAGA Reverse: CTTCCCTCTGTATACGCCATTGTAGC Probe: FAM-ATGGCTGTCGTCAGCTCGTGTT-BHQ1	[34]
*M. pneumoniae*	*P1*	Forward: CCAACCAAACAACAACGTTCA Reverse: ACCTTGACTGGAGGCCGTTA Probe: FAM-TCAATCCGAATAACGGTGACTTCTTACCACTG-BHQ1	[38]
*Legionella* spp.	*16SrRNA*	Forward: AGGCTAATCTTAAAGCGCCAGGCC Reverse: GCATGCTTAACACATGCAAGTCGAAC Probe: FAM-CATATTCCTACGCGTTACTCACCCGT-BHQ1	[34]
*P. aeruginosa*	*16SrRNA*	Forward: GACGGGTGAGTAATGCCTAGGA Reverse: CCACTGGTGTTCCTTCCTATATCT Probe: FAM-AGTGGGGGATCTTCGGACCTCA-BHQ1	[34]
*K. pneumoniae*	*gapA*	Forward: TGAAGTATGACTCCACTCACGGT Reverse: CTTCAGAAGCGGCTTTGATGGCTT Probe: FAM-CCGGTATCTTCCTGACCGACGA-BHQ1	[34]

**Table 4:** Performance of the MPLT assay compared to the real-time PCR assay.

Pathogens	Real-Time PCR Assay	MPLT Assay		Performance of the MPLT Assay			Measures of Agreement Kappa Values
		Positive	Negative	Consistency (%)	Sensitivity (%)	Specificity (%)	
*S. pneumoniae*	Positive	7	0	98.8	87.5	100	0.927 (*p* < 0.001)
	Negative	1	78				
*M. catarrhalis*	Positive	0	0	-	-	-	*
	Negative	0	86				
*S. aureus*	Positive	11	0	100	100	100	1.000 (*p* < 0.001)
	Negative	0	75				
*S. pyogenes*	Positive	0	0	-	-	-	*
	Negative	0	86				
*H. influenzae*	Positive	8	0	100	100	100	1.000 (*p* < 0.001)
	Negative	0	78				
*M. pneumoniae*	Positive	1	0	100	100	100	1.000 (*p* < 0.001)
	Negative	0	85				
*Legionella* spp.	Positive	0	0	-	-	-	*
	Negative	0	86				
*P. aeruginosa*	Positive	10	0	100	100	100	1.000 (*p* < 0.001)
	Negative	0	76				
*K. pneumoniae*	Positive	5	0	100	100	100	1.000 (*p* < 0.001)
	Negative	0	81				

* No statistics were computed because the MPLT assay and real-time PCR assay for *M. catarrhalis*, *S. pyogenes*, and *Legionella* spp. are constants.

## Supplementary Materials

The following are available online at www.mdpi.com/1660-4601/14/3/223/s1, Real-time PCR assays for detection of nine respiratory pathogens.

## Abbreviations

LRTI	lower respiratory tract infection
LRTIs	lower respiratory tract infections
MPLT	multiplex PCR and Luminex technology
PCR	polymerase chain reaction
BALF	bronchoalveolar lavage fluid
*S. pneumonia*	*Streptococcus pneumoniae*
*M. catarrhalis*	*Moraxella catarrhalis*
*S. aureus*	*Staphylococcus aureus*
*S. pyogenes*	*Streptococcus pyogenes*
*H. influenza*	*Haemophilus influenzae*
*M. pneumonia*	*Mycoplasma pneumoniae*
*P. aeruginosa*	*Pseudomonas aeruginosa*
*K. pneumonia*	*Klebsiella pneumoniae*
mPCR	multiplex PCR; SAPE, Streptavidin, R-Phycoerythrin Conjugate
MFI	median fluorescence intensity
cMFI	corrected MFI
SEM	standard error of the mean
CFU	colony forming unit
Ct	threshold cycle
