# Supplementary material for: Simultaneous Detection of Nine Key Bacterial Respiratory Pathogens Using Luminex xTAG® Technology
Source: Int J Environ Res Public Health. 2017 Feb 24;14(3):223. doi: 10.3390/ijerph14030223 (PMC5369059; doi:10.3390/ijerph14030223)
Supplement: Supplementary file 1 [file ijerph-14-00223-s001.pdf]

Supplementary Materials: Simultaneous Detection of Nine Key Bacterial Respiratory Pathogens using Luminex xTAG® Technology

Luxi Jiang, Hongyu Ren, Haijian Zhou, Tian Qin * and Yu Chen *

Real-time PCR assays for detection of nine respiratory pathogens

1. S. pneumoniae

1.1. Gene target: lytA

1.2. Oligonucleotide/Oligo Sequence

Forward: ACGCAATCTAGCAGATGAAGCA
Reverse: TCGTGCGTTTTAATTCCAGCT
Probe: FAM-GCCGAAAACGCTTGATACAGGGAG-BHQ1

1.3. Reaction system (total volume: 25μL)

Component	Volume
Forward primer (200nM)	1.25μL
Reverse primer (200nM)	1.25μL
Probe (200nM)	1.25μL
ROXII	0.5μL
distilled water	5.75μL
Premix Ex *Taq*	12.5μL
genomic DNA template	2.5μL

1.4. Amplification conditions

Temperature	Time	Cycles
95℃	10min	1 cycle
95℃	15s	40 cycles
60℃	1min	40 cycles

1.5. Results

bacterial concentration (CFU/mL)	Threshold cycle (Ct)	Ct	Ct	Ct
10^8	18.49	19.91	19.02	19.14±0.59
10^7	21.68	20.47	21.56	21.24±0.54
10^6	24.38	22.95	24.38	23.90±0.67
10^5	28.33	29.30	28.57	28.73±0.41
10^4	32.96	32.59	32.79	32.78±0.15
10^3	35.73	36.19	35.93	35.95±0.19
10^2	38.00	37.76	38.11	37.96±0.15
10^1	N	N	N	N
10^0	N	N	N	N

N: no Ct; Threshold cycle (Ct) for positive samples: ＜38.

2. M. catarrhalis

2.1. Gene target: copB

2.2. Oligonucleotide/Oligo Sequence

Forward: CGTGCGTGTTGACCGTTTTGACTTTA
Reverse: CACGCTGCCAAAAATAACTGCCAAAG
Probe: FAM-CAGCGGTAACCTAATCTATGCCACTC-BHQ1

2.3. Reaction system (total volume: 25μL)

Component	Volume
Forward primer (300nM)	1.25μL
Reverse primer (300nM)	1.25μL
Probe (300nM)	1.25μL
ROXII	0.5μL
distilled water	7.25μL
Premix Ex *Taq*	12.5μL
genomic DNA template	1μL

2.4. Amplification conditions

Temperature	Time	Cycles
95℃	30s	1 cycle
95℃	8s	40 cycles
61℃	25s	40 cycles
72℃	20s	40 cycles

2.5. Results

bacterial concentration (CFU/mL)	Ct	Ct	Ct	Ct
10^8	20.77	19.88	21.16	20.60±0.54
10^7	23.52	22.38	22.61	22.84±0.49
10^6	27.59	26.00	27.25	26.95±0.68
10^5	29.18	30.61	29.87	29.89±0.58
10^4	32.74	31.64	33.78	32.72±0.87
10^3	36.90	36.19	36.70	36.60±0.30
10^2	38.41	37.66	38.43	38.17±0.36
10^1	N	N	N	N
10^0	N	N	N	N

N: no Ct; Threshold cycle (Ct) for positive samples: ＜38.

3. S. aureus

3.1. Gene target: spa

3.2. Oligonucleotide/Oligo Sequence

Forward: TACATGTCGTTAAACCTGGTG
Reverse: TACAGTTGTACCGATGAATGG
Probe: FAM-CGCGATCCAAGAACTTGTTGTTGATAAGAAGCAACCGATCGCG-BHQ1

3.3. Reaction System (total volume: 25μL)

Component	Volume
Forward primer (200nM)	1.25μL
Reverse primer (200nM)	1.25μL
Probe (300nM)	1.25μL
ROXII	0.5μL
distilled water	7.25μL
Premix Ex *Taq*	12.5μL
genomic DNA template	1μL

3.4. Amplification Conditions

Temperature	Time	Cycles
95℃	30s	1 cycle
95℃	15s	40 cycles
50℃	30s	40 cycles
75℃	30s	40 cycles

3.5. Results

bacterial concentration (CFU/mL)	Ct	Ct	Ct	Ct
10^8	23.53	23.54	23.68	23.58±0.07
10^7	26.50	26.03	27.18	26.57±0.47
10^6	30.89	30.36	30.92	30.72±0.26
10^5	34.96	34.75	35.21	34.97±0.19
10^4	36.76	36.59	37.51	36.95±0.40
10^3	N	38.56	38.87	38.72±0.16
10^2	N	N	N	N
10^1	N	N	N	N
10^0	N	N	N	N

N: no Ct; Threshold cycle (Ct) for positive samples: ＜38.

4. S. pyogenes

4.1. Gene target: 16SrRNA

4.2. Oligonucleotide/Oligo Sequence

Forward: GAGAGACTAACGCATGTTAGTA
Reverse: TAGTTACCGTCACTTGGTGG
Probe: FAM-CGCGATCGCGACGATACATAGCCGACCTGGATCGCG-BHQ1

4.3. Reaction System (total volume: 25μL)

Component	Volume
Forward primer (150nM)	1.25μL
Reverse primer (150nM)	1.25μL
Probe (300nM)	1.25μL
ROXII	0.5μL
distilled water	7.25μL
Premix Ex *Taq*	12.5μL
genomic DNA template	1μL

4.4. Amplification Conditions

Temperature	Time	Cycles
95℃	30s	1 cycle
95℃	15s	40 cycles
50℃	30s	40 cycles
75℃	30s	40 cycles

4.5. Results

bacterial concentration (CFU/mL)	Ct	Ct	Ct	Ct
10^8	18.82	18.95	18.85	18.87±0.06
10^7	20.68	21.79	20.80	21.09±0.50
10^6	24.44	25.70	25.60	25.25±0.57
10^5	29.61	29.79	29.91	29.77±0.12
10^4	32.03	32.69	33.25	32.66±0.50
10^3	36.51	36.46	36.57	36.51±0.04
10^2	N	38.46	38.03	38.25±0.22
10^1	N	N	N	N
10^0	N	N	N	N

N: no Ct; Threshold cycle (Ct) for positive samples: ＜38.

5. H. influenzae

5.1. Gene Target: 16SrRNA

5.2. Oligonucleotide/Oligo Sequence

Forward: TTGACATCCTAAGAAGAGCTCAGAGA
Reverse: CTTCCCTCTGTATACGCCATTGTAGC
Probe: FAM-ATGGCTGTCGTCAGCTCGTGTT-BHQ1

5.3. Reaction System (total volume: 25μL)

Component	Volume
Forward primer (300nM)	1.25μL
Reverse primer (300nM)	1.25μL
Probe (300nM)	1.25μL
ROXII	0.5μL
distilled water	7.25μL
Premix Ex *Taq*	12.5μL
genomic DNA template	1μL

5.4. Amplification Conditions

Temperature	Time	Cycles
95℃	30s	1 cycle
95℃	8s	40 cycles
61℃	25s	40 cycles
72℃	20s	40 cycles

5.5. Results

**bacterial concentration (CFU/mL)**	**Ct**	**Ct**	**Ct**	**Ct**
10^8	16.41	16.42	16.46	16.43±0.02
10^7	19.36	19.84	19.59	19.60±0.20
10^6	23.26	22.70	21.91	22.62±0.55
10^5	26.12	25.92	25.88	25.97±0.10
10^4	29.52	29.63	29.60	29.58±0.05
10^3	33.01	33.16	33.21	33.13±0.08
10^2	36.54	36.00	36.98	36.51±0.40
10^1	N	N	N	N
10^0	N	N	N	N

N: no Ct; Threshold cycle (Ct) for positive samples: ＜36.

6. M. pneumoniae

6.1. Gene Target: P1

6.2. Oligonucleotide/Oligo Sequence

Forward: CCAACCAAACAACAACGTTCA
Reverse: ACCTTG ACT GGA GGC CGT TA
Probe: FAM-TCAATCCGAATAACGGTGACTTCTTACCACTG-BHQ1

6.3. Reaction System (total volume: 25μL)

**Component**	**Volume**
Forward primer (100nM)	1.25μL
Reverse primer (100nM)	1.25μL
Probe (50nM)	1.25μL
ROXII	0.5μL
distilled water	7.25μL
Premix Ex *Taq*	12.5μL
genomic DNA template	1μL

6.4. Amplification Conditions

**Temperature**	**Time**	**Cycles**
50℃	2min	1 cycle
95℃	10min	1 cycle
95℃	15s	45 cycles
60℃	1min	45 cycles

6.5. Results

**concentration of DNA (ng/μL)**	**Ct**	**Ct**	**Ct**	**Ct**
10^1	16.20	16.47	16.67	16.45±0.19
10^0	20.14	20.11	21.87	20.71±0.82
10^{-1}	23.45	23.34	23.25	23.35±0.08
10^{-2}	26.82	26.59	26.58	26.66±0.11
10^{-3}	29.52	30.00	30.04	29.85±0.24

10^{-4}	33.01	33.45	33.13	33.20±0.19
10^{-5}	36.15	36.00	36.96	36.37±0.42
10^{-6}	N	N	39.94	N

N: no Ct; Threshold cycle (Ct) for positive samples: ＜36.

7. Legionella spp.

7.1. Gene Target: *16SrRNA*

7.2. Oligonucleotide/Oligo Sequence

Forward: AGGCTAATCTTAAAGCGCCAGGCC
Reverse: GCATGCTTAACACATGCAAGTCGAAC
Probe: FAM-CATATTCCTACGCGTTACTCACCCGT-BHQ1

7.3. Reaction System (total volume: 25μL)

Component	Volume
Forward primer (300nM)	1.25μL
Reverse primer (300nM)	1.25μL
Probe (100nM)	1.25μL
ROXII	0.5μL
distilled water	7.25μL
Premix Ex *Taq*	12.5μL
genomic DNA template	1μL

7.4. Amplification Conditions

Temperature	Time	Cycles
95℃	30s	1 cycle
95℃	8s	40 cycles
61℃	25s	40 cycles
72℃	20s	40 cycles

7.5. Results

bacterial concentration (CFU/mL)	Ct	Ct	Ct	Ct
10^{8}	15.12	15.25	16.00	15.46±0.39
10^{7}	17.31	18.52	18.34	18.06±0.53
10^{6}	21.16	21.81	21.35	21.44±0.27
10^{5}	24.81	24.93	23.76	24.50±0.53
10^{4}	28.72	28.27	28.87	28.62±0.25
10^{3}	31.99	31.63	32.51	32.04±0.36
10^{2}	36.57	36.12	35.89	36.19±0.28
10^{1}	N	N	N	N
10^{0}	N	N	N	N

N: no Ct; Threshold cycle (Ct) for positive samples: ＜36.

8. P. aeruginosa

8.1. Gene target: 16SrRNA

8.2. Oligonucleotide/Oligo Sequence

Forward: GACGGGTGAGTAATGCCTAGGA
Reverse: CCACTGGTGTTCCTTCCTATATCT
Probe: FAM-AGTGGGGGATCTTCGGACCTCA-BHQ1

8.3. Reaction System (total volume: 25μL)

Component	Volume
Forward primer (300nM)	1.25μL
Reverse primer (300nM)	1.25μL
Probe (300nM)	1.25μL
ROXII	0.5μL
distilled water	7.25μL
Premix Ex *Taq*	12.5μL
genomic DNA template	1μL

8.4. Amplification Conditions

Temperature	Time	Cycles
95℃	30s	1 cycle
95℃	8s	40 cycles
61℃	25s	40 cycles
72℃	20s	40 cycles

8.5. Results

bacterial concentration (CFU/mL)	Ct	Ct	Ct	Ct
10^8	17.27	17.33	17.44	17.35±0.07
10^7	20.50	20.57	20.45	20.51±0.05
10^6	23.42	23.57	24.05	23.68±0.27
10^5	26.63	26.61	27.28	26.84±0.31
10^4	30.01	30.27	30.32	30.20±0.14
10^3	34.11	33.16	33.83	33.70±0.40
10^2	N	35.47	35.89	35.68±0.21
10^1	N	N	N	N
10^0	N	N	N	N

N: no Ct; Threshold cycle (Ct) for positive samples: ＜35.

9. K. pneumoniae

9.1. Gene Target: gapA

9.2. Oligonucleotide/Oligo Sequence

Forward: TGAAGTATGACTCCACTCACGGT
Reverse: CTTCAGAAGCGGCTTTGATGGCTT
Probe: FAM-CCGGTATCTTCCTGACCGACGA-BHQ1

9.3. Reaction System (Total Volume: 25μL)

Component	Volume
Forward primer (300nM)	1.25μL
Reverse primer (300nM)	1.25μL
Probe (300nM)	1.25μL
ROXII	0.5μL
distilled water	7.25μL
Premix Ex *Taq*	12.5μL
genomic DNA template	1μL

9.4. Amplification Conditions

Temperature	Time	Cycles
95℃	30s	1 cycle
95℃	8s	40 cycles
61℃	25s	40 cycles
72℃	20s	40 cycles

9.5. Results

bacterial concentration (CFU/mL)	Ct	Ct	Ct	Ct
10^8	18.63	17.52	18.81	18.32±0.57
10^7	20.38	19.08	20.46	19.97±0.63
10^6	23.51	22.64	23.39	23.18±0.38
10^5	27.47	27.39	27.20	27.35±0.11
10^4	30.01	31.54	30.30	30.62±0.66
10^3	34.22	34.99	35.03	34.75±0.37
10^2	36.00	37.05	38.40	37.15±0.98
10^1	N	N	N	N
10^0	N	N	N	N

N: no Ct; Threshold cycle (Ct) for positive samples: ＜37.

© 2017 by the authors; licensee MDPI, Basel, Switzerland. This article is an open access article distributed under the terms and conditions of the Creative Commons by Attribution (CC-BY) license (http://creativecommons.org/licenses/by/4.0/).
